# Focal adhesion kinase inhibitors, a heavy punch to cancer

**DOI:** 10.1007/s12672-021-00449-y

**Published:** 2021-11-22

**Authors:** Yueling Wu, Ning Li, Chengfeng Ye, Xingmei Jiang, Hui Luo, Baoyuan Zhang, Ying Zhang, Qingyu Zhang

**Affiliations:** 1 Department of Obstetrics and Gynecology, Affiliated Hospital of Guangdong Medical University, Zhanjiang, 524001 China; 2 Graduate School of Guangdong Medical University, Zhanjiang, 524023 China; 3 The Marine Biomedical Research Institute, Guangdong Medical University, Zhanjiang, 524023 China; 4 Shanghai Institute of Hematology, State Key Laboratory for Medical Genomics, Ruijin Hospital, Shanghai Jiao Tong University School of Medicine, Shanghai, 200025 China

**Keywords:** Focal adhesion kinase, FAK inhibitors, Cancer chemotherapy

## Abstract

Kinases are the ideal druggable targets for diseases and especially were highlighted on cancer therapy. Focal adhesion kinase (FAK) is a non-receptor tyrosine kinase and its aberrant signaling extensively implicates in the progression of most cancer types, involving in cancer cell growth, adhesion, migration, and tumor microenvironment (TME) remodeling. FAK is commonly overexpressed and activated in a variety of cancers and plays as a targetable kinase in cancer therapy. FAK inhibitors already exhibited promising performance in preclinical and early-stage clinical trials. Moreover, substantial evidence has implied that targeting FAK is more effective in combination strategy, thereby reversing the failure of chemotherapies or targeted therapies in solid tumors. In the current review, we summarized the drug development progress, chemotherapy strategy, and perspective view for FAK inhibitors.

## Background

Focal adhesion kinase (FAK), encoded by the *PTK2* gene (protein tyrosine kinase 2), is a non-receptor tyrosine kinase. It is well known that FAK is phosphorylated and activated by integrins or growth factors, which transduce extracellular signals into cells to response the dynamic changes in microenvironment [1, 2]. Extracellular matrix can support the tumorigenicity and the disease progression. Specifically, Integrin acts as the anchor of cells to its adjacent matrix components, and Integrin/FAK as a signaling bridge to connect the tumor microenvironment and the cancer cells, which helps the deteriorated cells to acclimate the cancer-associated contexts. FAK is ubiquitously overexpressed in a series of cancer types, consisting of breast, oral, colon, gastric, and ovarian cancers as well as hepatocellular carcinoma [3–5]. FAK exerts its functions through the phosphorylation on the corresponding downstream target proteins in the cytoplasm, thereby enhancing tumor cell adhesion and promoting tumor growth, cancer-stemness, invasion, and metastasis ability [6, 7], promoting cancer cell epithelial to mesenchymal transition (EMT), tumor angiogenesis, chemotherapeutic resistance, and fibrosis in the stroma. Besides, regulation of target protein function through FAK scaffolding activity also contributes to cancer progression [8]. Furthermore, FAK kinase quick response to cancer drug treatment predicate FAK kinase exerts a positive effect in cancers [8]. Therefore, inhibition of FAK kinase activity may effectively suppress tumor proliferation, metastasis, and chemo-resistance, and is expected to serve as a qualified strategy for cancers treatment. In this review, we briefly summarized the structure and functions of FAK and mainly introduced the research progress of FAK inhibitors regarding cancer treatment in the past decades.

## FAK structural features and the function in cancer

**Fig. 1:**
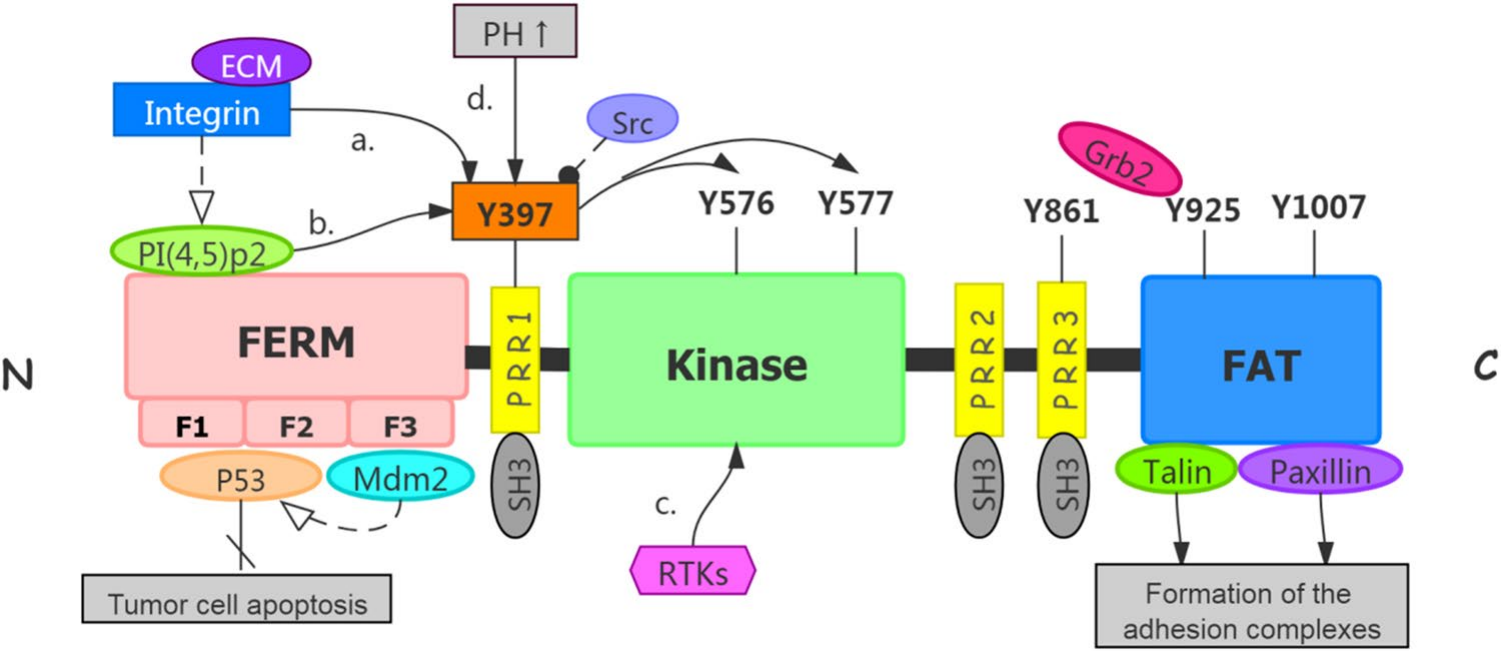
structure of FAK protein and activation of FAK. 1.1 The protein structure of FAK which contains three major domains and three PPR small domains between the three major domains. 1.2 The activation of FAK. **a** Integrin binding to the relevant ligand on the extracellular matrix leads to Tyr397 autophosphorylation of FAK and FAK activation [16]. **b** Phosphatidylinositol 4,5-bisphosphate [PI(4,5)P2] binds to FERM mediated by integrins, the Tyr397 phosphorylation site is exposed and autophosphorylated [17]. Tyr397 phosphorylation recruits Src, which further phosphorylates Tyr576 and Tyr577 to release kinase domain from PERM domain, to make FAK reaches a fully activated state. **c** The receptor tyrosine kinases (RTKs) can directly activate the phosphorylation activation loop in FAK kinase region, thereby upregulating FAK kinase activity [18]. **d** Elevation pH reduces the stability of the FERM/kinase region interaction, resulting in phosphorylation of Y397 [19]. Activated FAK arrested p53 or convene Mdm-2 to enhance ubiquitination of p53 to block apoptosis in tumor cells. Talin and Paxillin bind to integrins in cytoplasmic regions, which can mediate the formation of adhesion complexes [1].

FAK is a 125 kDa protein consisting of three linearly arranged structural domains, including a trilobular structure N-terminus FERM structural domain located at the N-terminus. The FAT domain is located at the C-terminus. The catalytic kinase structural domain located between the FERM domain and FAT domain. Both terminus domains are separated from the kinase domain by linker regions that contain proline-rich regions (PRRs). (Fig. 1). It has been demonstrated that FAK contains multiple functional phosphorylation sites (Tyr397, Tyr407, Tyr576, Tyr577, Tyr861, Tyr925), of which Tyr397 is one of the most important phosphorylation site [9] and interacts directly with the Src family. Tyr576 and 577 are in the activation loop of the kinase domain, which are the main sites can be phosphorylated by Src family [9]. Tyr925 is phosphorylated and binds to the junction protein Grb2 to make FAK aggregate with integrins, while Tyr397 and Tyr 861 both could recruit other SH2 proteins [10].

### N-terminus FERM domain

There are multiple protein binding sites for signal transduction proteins, cytoskeleton proteins, and integrin β subunits at the N-terminus. The main functional domain of this structural domain is FERM (four-point-one, ezrin, radixin, moesin) domain, which contains three closely related subdomains (F1, F2, F3), forming a cloverleaf shape [11]. Among them, the F1 and F2 subdomains interact with p53 to block apoptosis in tumor cells. The F2 subdomain regulates the kinase-independent activity and mediates cell survival. The F3 subdomain can arrest Mdm-2 to enhance ubiquitination of p53, which alleviates p53 independent cell apoptosis [11, 12]. In addition, the key tyrosine residue, Tyr397 is located at the N-terminus end of the FERM structural domain. Tyr397 autophosphorylation generates a high-affinity binding site for SH2 rich protein recruitment and subsequent activation of the FAK downstream pathway [13].

### Central kinase domain

The central kinase domain has an activation loop that contains two important Tyr sites at576 and 577 which can be phosphorylated by Src and stimulate FAK kinase activity in turn [14]. The classic mechanism of FAK activation involves integrin receptor clustering upon the binding of cells to extracellular matrix (ECM) proteins, which leads to FAK autophosphorylation at Tyr397. FAK autophosphorylation at Tyr397 recruit Src-family kinases to phosphorylate FAK kinase activation loop atTyr576 and Tyr577, and finally formed a fully active FAK-SRC complex. Therefore, Tyr397 is one important phosphorylation located between FERM and the kinase region. Its autophosphorylation modulates the activity of FAK and consequently affects the biological functions and cell behaviors. Active FAK can phosphorylate various proteins such as the Src family, phospholipase C7, SHC adaptor protein, and growth factor connector protein 7 etc. [15].

### C-terminus bomain

The C-terminus structural domain comprises two proline-rich regions (PRR2 and PRR3) and the Focal adhesion targeting (FAT) domain. Like the N-terminus FERM structural domain, the C-terminus region is also involved in various protein interactions. The C-terminus FAT is a functional domain for FAK adhesion to adhesive patches. The FAT domain contains binding sites for adhesion-associated proteins (such as paxillin and talin) that bind directly to integrins in the cytoplasmic region, thereby mediating the formation of the adhesion complex. In contrast, PRR2 and PRR3 at the C-terminus provide direct binding sites for proteins containing SH3 structures. Tyr861 and Tyr925 can be phosphorylated to form binding sites for proteins containing the SH2 structural domain. Thus, the C-terminus structural domain is also involved in regulating endogenous FAK function [14, 15].

### The role of FAK in the cancer cell and tumor microenvironment remodeling

**Fig. 2:**
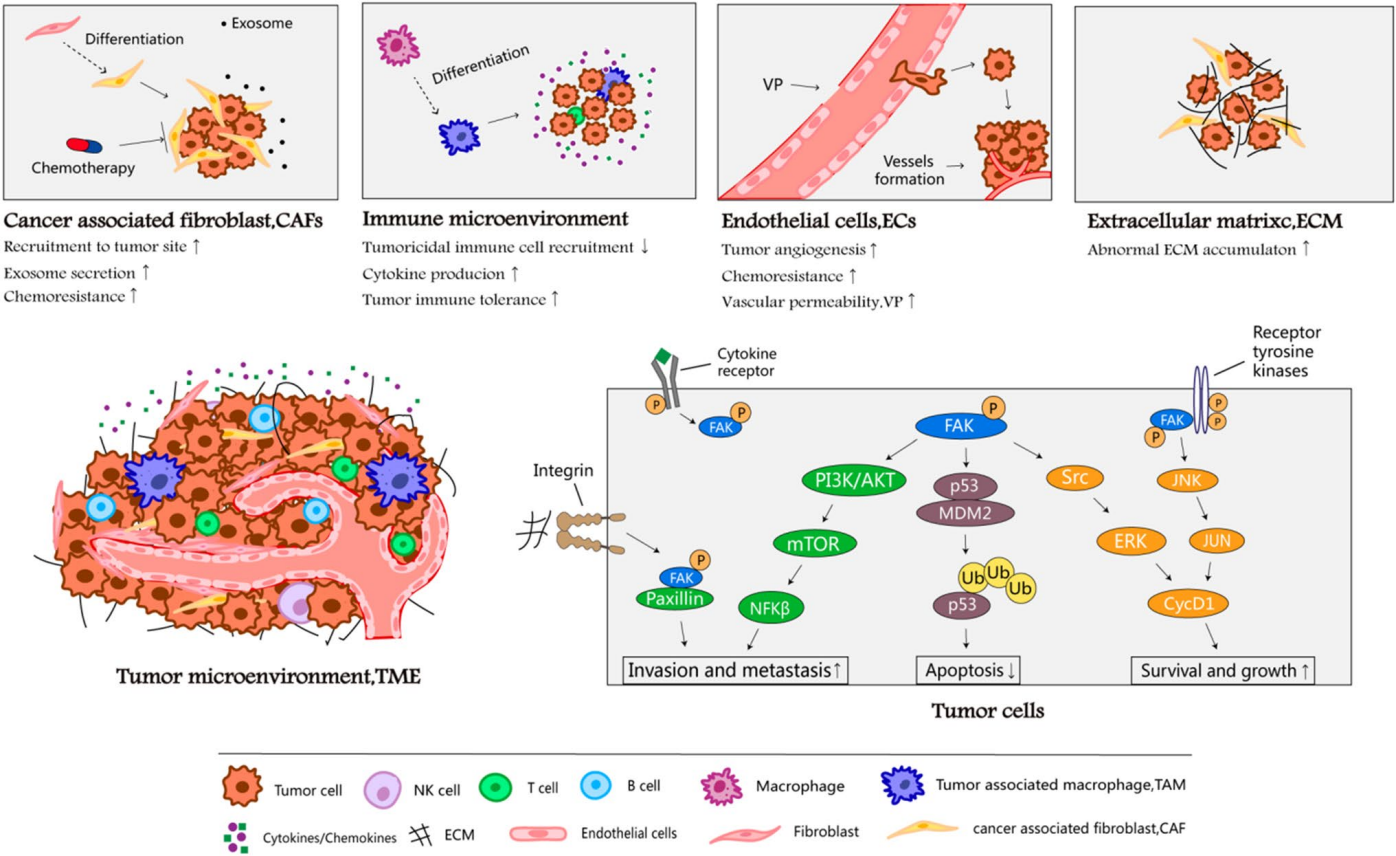
Multiple role of FAK in maintaining cancer malignancy. FAK kinase activation not only triggers ovarian cancer malignancy but also resulted in the recruitment of tumor-related cells including cancer-associated fibroblast, immune cells, endothelial cells as well as extracellular matrix remolding. FAK multiple roles in tumor progression evolution promise FAK inhibitor potential in mitigating tumor overgrowth, chemotherapy resistance, and immune escape

FAK regulation of cancer progression through “self-activation” and microenvironment remodeling for tumor seed preservation and niche cultivation (Fig. 2). Specifically, FAK activates FAK/PI3K, FAK/MAPK, FAK/p53, and other pathways related to cell growth, survival, and apoptosis [20]. Recent studies have demonstrated the role of FAK in promoting TME remodeling. In tumor-associated endothelial cells, FAK expression and phosphorylation levels of Tyr397 were elevated [21]. It was noted that stimulatory changes in EC migration are an essential component of angiogenesis, and that FAK activation downstream of growth factor, integrin, and cytokine receptors contributes to EC motility [22, 23]. Huang et al. showed that ECs acquire transformation into a mesenchymal stem cell (MSC)-like cells in glioblastoma (GBM), thus driving tumor resistance to cytotoxic therapy [24]. Furthermore, Jean et al. pointed out that FAK inhibition reduced tumor angiogenesis in the animal model of human ovarian cancer, indicating the positive role of FAK in angiogenesis [25].

FAK, on the other hand, allows tumor cells to compromise with host immune cells in TME and evade the immune surveillance by recruiting immunosuppressive cells or secreting cytokines, cell immunity modulation [26]. Walsh et al. reported that the use of FAK inhibitors reduced leukocytes and macrophages infiltration and reduced tumor growth in a mammary carcinoma mouse model [27, 28]. Stokes et al. verified that pharmacological inhibition of FAK reduced TAMs within the tumor and reduced the size of the primary tumor in a pancreatic ductal adenocarcinoma mouse model [29].

Moreover, upon FAK activation, the ECM plays an important role in tumor progression by providing tumor cells with sustained proliferative signals, forming desmoplastic stroma, and evading growth inhibitory factors [30]. Finally, similar to TAMs, FAK inhibition in a bleomycin-induced fibrosis mouse model shows marked abrogation of lung fibrosis [31]. Overall, there is growing evidence for FAK, as a regulator of ECs, macrophage, and fibroblast signaling in TME, promotes the remodeling tumor microenvironment.

## The development of FAK inhibitors

As reviewed above that FAK plays a vital role in many facets of tumors, and a consensus was widely reached in the science community that FAK is a promising target for the development of anti-cancer drugs. Many small-molecule FAK inhibitors have been developed and some even put forward to clinical trials ongoing or done. In this section, we summarized the progress in the development of FAK inhibitors.

### ATP-competitive FAK inhibitors

Over the past decades, multiple preclinical and clinical-stage FAK inhibitors have been assessed for their effects in treating cancer diseases [9, 15, 32, 33]. Table 1 summarized five oral ATP-competitive FAK inhibitors that have been evaluated in clinical trials.

**Table 1:** Clinical trials with ATP-competitive FAK inhibitors

FAK inhibitor name	Combination agents	Cancer type	Phase	Status	NCI identifier
VS-6062 (PF00562271)	/	Pancreatic, head and neck, prostatic neoplasms	Phase I	Completed [45]	NCT00666926
VS-6063 (Defactinib)	/	Non-hematologic malignancies	Phase I	Completed [46]	NCT00787033
	/	Non-hematologic cancers	Phase I	Completed	NCT01943292
	/	Healthy subjects	Phase I	Completed	NCT02913716
	/	Non-small cell lung cancer	Phase II	Completed [47]	NCT01951690
	Paclitaxel, Defactinib	Ovarian cancer	Phase I	Completed	NCT01778803
	RO5126766	NSCLC, LGSOC Colorectal cancer	Phase I	Recruiting	NCT03875820
	VS-6766	Ovarian cancer	Phase II	Recruiting	NCT04625270
	VS-6766	Non-small cell lung cancer	Phase II	Recruiting [48]	NCT04620330
	VS-6766	Metastatic uveal melanoma	Phase II	Recruiting	NCT04720417
	VS-6766	Ovarian cancer	Phase II	Recruiting	NCT04625270
	Pembrolizumab	Pancreatic ductal Adenocarcinoma	Phase II	Recruiting	NCT03727880
	Pembrolizumab	Mesothelioma	Phase I Phase II	Recruiting	NCT02758587
	Paclitaxel, Carboplatin	Ovarian cancer	Phase I Phase II	Recruiting	NCT03287271
	/	Advanced lymphoma	Phase II	Active, not recruiting	NCT04439331
	PembrolizumabGemcitabine	Advanced solid tumors	Phase I	Active, not recruiting	NCT02546531
	Pembrolizumab	Malignant pleural mesothelioma	Phase I	Withdrawn	NCT04201145
	Avelumab	Epithelial ovarian cancer	Phase I	Terminated	NCT02943317 [49]
	/	Malignant pleural mesothelioma	Phase II	Terminated	NCT02004028
	Placebo	Malignant pleural mesothelioma	Phase II	Terminated [50]	NCT01870609
GSK2256098	Trametinib	Pancreatic cancer Adenocarcinoma	Phase II	Recruiting	NCT02428270
	Trametinib	Neoplasms	Phase I	Completed [51]	NCT01938443
	Placebo	Healthy volunteers	Phase I	Completed	NCT00996671
	/	Solid tumors	Phase I	Completed [52]	NCT01138033
	Vismodegib	Meningioma	Phase II	Suspended	NCT02523014
PND-1186 (VS-4718)	Nab-Paclitaxel, Gemcitabine	Pancreatic cancer	Phase I	Terminated	NCT02651727
	/	Non-hematologic cancers	Phase I	Terminated	NCT01849744
	/	Relapsed or refractory acute myeloid leukemia	Phase I	Withdrawn	NCT02215629 [53]
IN10018 (BI-853520)	Cobimetinib	Metastatic melanoma	Phase I	Recruiting	NCT04109456
	Traditional chemotherapy	HGSOC Oviduct cancer	Phase I	Recruiting	CTR20200913
	Docetaxel	Gastric cancer	Phase I	Recruiting	CTR20192715
	/	Neoplasms	Phase I	Completed [54]	NCT01905111
	/	Neoplasms	Phase I	Completed	NCT01335269

#### TAE226

TAE226, also known as NVP-226, inhibits FAK activity by blocking the linkage between FAK phosphorylation sites of Tyr397 and Tyr861 and ATP binding pocket. TAE226 has shown anti-tumor effects in preclinical in vivo and in vitro assays in non-small cell lung cancer (NSCLC) [34], Ewing’s Sarcoma (EWS) [35], Ph^+^Acute lymphoblastic leukemia (Ph^+^ALL) [36], oral squamous cell carcinoma (OSCC) [37], colorectal carcinoma [38] and pancreatic ductal adenocarcinoma (PDAC) [39]. Nevertheless, TAE226 had not been approved for clinical trials due to serious side effects on glucose metabolism [40].

#### VS-6062

VS-6062, also known as PF-562271 or PF00562271, is an orally administered biological agent that is a potent dual ATP-competitive inhibitor of FAK and FAK2. It blocks the phosphorylation of FAK Tyr397 and inhibits FAK overexpression in a dose-dependent manner, resulting in antitumor effects in rhabdomyosarcoma (RMS) [41], epithelial ovarian cancer (EOC) [42, 43] and PDAC [29]. Phase I clinical trials (NCT00666926) of VS-6062 have been completed as the first specific FAK inhibitor in clinical trials (head and neck cancer, prostate cancer and pancreatic cancer). This clinical trial has confirmed that VS-6062 has low toxicity and potent tumor-suppressive effects, toxicities included headache, nausea, vomiting, dehydration and edema [44].

#### PF-573228

PF-573228, also known as PF-228, which is current in preclinical studies, effectively blocks the phosphorylation of FAK Tyr397. PF-573228 can not only inhibit FAK signaling but also arrest tumor growth and invasion in bladder cancer [55], hemangioma [56], small cell lung cancer(SCLC) [57] and Neuroblastoma [58]. Meanwhile, tumor suppression and prolonged survival were also observed in animal models [55].

#### VS-6063

VS-6063, also known as PF-04554878 and defactinib, is a second-generation FAK inhibitor by suppressing of FAK Tyr397 phosphorylation. In detail, defactinib is now studied in nineteen clinical trials, five of which completed (NCT00787033, NCT01943292, NCT02913716, NCT01951690, NCT01778803), eight in recruiting status and two actives, but not yet recruiting. Ten are phases I studies, one of them in healthy patients, others eight are phase II studies. The phase I clinical trials in non-hematologic malignancies had been completed [46], which demonstrated a favorable and safe profile in these patients even including advanced solid tumors. The phase II clinical trials for Defactinib (VS-6063) have been completed in patients with KRAS mutant non-small cell lung cancer (NSCLC) and the drug was generally well tolerated and suitable for long-term dosing [59]. The results of these clinical trials suggest that the most common adverse events were nausea, vomiting, unconjugated hyperbilirubinemia, fatigue, headache, diarrhea [46, 47, 50, 60, 61].

Moreover, one study (NCT01951690) [61] on NSCLC patients harboring KRAS mutation was discontinued due the death of 76% of patients, in addition another study failed phase II multicenter clinical trial (NCT01870609) targeting malignant pleural mesothelioma stem cells [62]. There are still eight Phase I or II clinical trials under recruitment to evaluate the antineoplastic potential in ovarian cancer, non-small cell lung cancer and melanoma [63].

#### GSK2256098

GSK2256098 is an inhibitor targeting the FAK Tyr397 site In recent years, preclinical studies on GSK2256098 have found that it inhibits cell proliferation, migration and invasion of renal cell carcinoma [64], uterine cancer [65] and pancreatic ductal adenocarcinoma [66], and leads to lower tumor weight and less metastasis in mouse models. GSK2256098 is now studied in five clinical trials, three of which completed (NCT01938443, NCT00996671, NCT01138033). Preliminary results show that GSK2256098 was well tolerated with mild nausea, diarrhea, vomiting, decreased appetite and asthenia. A Phase II clinical trial (NCT02428270) in combination with trametinib for advanced pancreatic ductal carcinoma is in recruitment [67], The study is designed to evaluate the antitumor activity of GSK2256098 and Trametinib in patients with advanced pancreatic cancer, and commenced in April, 2016 and is expected to complete in December, 2022.

#### VS-4718

VS-4718, a potent reversible inhibitor of FAK, also known as PND-1186. An initial preclinical study by Buggio et al. indicated that VS-4718 monotherapy reduced the proliferation and increased the apoptosis of MM cells [68]. Jiang et al. reported that the single-dose FAK inhibition of VS-4718 greatly obstacles the tumor progression, increases two-folds survival rate in a human LSL-Kras (G12D) KPC mouse model [69]. Nevertheless, regarding the fact that all three of its clinical trials were terminated or withdrawn, the company did not announce the reasons [53].

#### IN10018

IN10018, also known as BI-853520, is a potent FAK inhibitor that inhibits the catalytic activity of FAK. IN10018 has been verified to inhibit Tyr397 phosphorylation in a series of human cancers and suppress tumor growth and progression in multiple mouse models of different cancer types [70]. In addition, IN10018 showed a manageable safety profile from a phase I study in Japanese and Taiwanese patients with solid tumors [54]. Six patients (29%) achieved a complete response (CR), suggesting that IN10018 exhibits good antitumor activity. IN10018 will soon enter into phase I clinical trials, including IN10018 in combination with cobimetinib for metastatic melanoma, with conventional chemotherapy for advanced plasma cancer, and with Docetaxel for gastric cancer [71, 72].

### Specific FAK inhibitors

Specific FAK inhibitors that bind to distinct kinase domain sites and do not directly compete with ATP binding are being developed in recent years [73, 74]. These FAK inhibitors (C4, Y11, Y15, and R2) have the potential for high FAK specificity, they show anti-tumor activity in cells and xenograft mouse models. Moreover, they have been reported can enhance the anti-tumor activity of other chemotherapeutics but have not been rigorously tested in clinical trials.

#### Y11

Y11 is a small molecule inhibitor of FAK designed by computer modeling combined with a functional assay approach, which directly bound to the N-terminus domain of FAK to preventTyr397 autophosphorylation. The in vitro tests showed that Y11 significantly decreased Tyr397 phosphorylation producing cell growth inhibition of colon cancer cell line SW620 and breast cancer cell line BT474 and also showed tumor growth inhibition for a colon cancer xenograft model [75].

#### Y15

Y15 was directly bound to FAK phosphorylated site Tyr397 in the FERM structural domain and inhibited Tyr397 phosphorylation in a time and dose-dependent manner. Y15 did not target homologous Pyk-2, c-Src, c-RAF, EGFR, IGFR, PDGFR, PI3K, VEGFR-3, and c-Met [76]. From in vitro studies, Y15 significantly inhibited cancer cell viability in six cancer cell lines, including breast cancer, thyroid cancer, colon cancer [77] glioblastoma tumor [78], Lung cancer [79] and Ewing’s sarcoma [80]. Further evidence indicated that Y15 promoted the pancreatic cancer cells apoptosis and inhibited the cell adhesion in a dose-dependent manner [81]. From in *vivo* assessment, Y15 was effective in causing regression of pancreatic cancer, inducing synergistic effects when combined with Gemcitabine [81].

#### C4

C4 inhibits FAK activity by hampering the interaction of the C-terminus region of FAK and is currently developed with preclinical experiments [82] C4 treatment resulted in FAK inactivation, reduced cell viability and proliferation, cell cycle arrest and apoptosis in pancreatic cancer cells. Mechanismly, C4 highly specific disrupt FAK-VEGFR3 interactions resulted in cell cycle arrest [83]. C4 increased the sensitivity of cancer cells to Gemcitabine in vitro and inhibited tumor growth in vivo [84].

#### R2

R2 Compound can specifically disrupt the FAK-p53 interaction by specifically blocking their binding site, increase the transcriptional activity of p53, which is currently assessed in clinical trials. R2 reduced the tumor volume of HCT116 colon cancer model. Notably, the efficacy of R2 in treating colon cancer was even better than that of standardized treatments. It also showed synergistic anticancer effects when combined with 5-fluorouracil or doxorubicin [85].

### FAK inhibitors in combination with anti-cancer drugs improves efficacy

Over the past few decades, treatment modalities for metastatic cancer cells have evolved from cytotoxic chemotherapy to targeted therapies. Therapeutic interventions combined with multiple target anti-cancer agents against different but interrelated tumorigenic mechanisms are more likely to eliminate cancer cells and reduce the likelihood of drug resistance development. For example, VEGF inhibitor and carboplatin separately target tumor angiogenesis and cell DNA replication, but their combination showed a synergic effect. Ongoing clinical trials, supported by in vitro and in vivo experimental studies, suggests that cytotoxic drugs are more effective in combination with some specific targeted therapies.

This section described studies of ATP-competitive FAK inhibitors (Table 2) and specific FAK inhibitors (Table 3) in combination with other anti-cancer drugs in vivo and in vitro over the past 5 years.

**Table 2:** Combination agents of ATP-competitive FAK inhibitor

FAK inhibitor name	Combination agents	Cancer Type	In vitro experiments		In vivo experiments	
			Synergistic effects	Reverse resistance	Inhibit growth	Extended survival
TAE226 (NVP-226)	Docetaxel	OC	√	√	√	√
	Conventional chemotherapeutic	EWS	√			
	Nilotinib	Ph^+^ALL	√		√	
	(nab-)paclitaxel	PDAC	√		√	√
VS-6062 (PF00562271)	AZD-1152	EWS	√		√	√
	Ganciclovir	GBM	√		√	
	ABT-737	OCCC	√			√
PF-573228 (PF-228)	Erlotinib	NSCLC	√	√		
	Lexatumumab	PDAC	√	√	√	
	Tamoxifen	ER^+^BC	√			
VS-6063 (Defactinib)	Docetaxel	CRPC	√		√	
	Paclitaxel	OC	√	√	√	√
	Gefitinib	NSCLC	√	√	√	
	(nab-)paclitaxel	PDAC	√		√	
	Everolimus	PanNETs	√		√	
GSK2256098	Gemcitabine	PC	√	√		√
	Paclitaxel	Uterine cancer	√		√	
VS-4718 (PND-1186)	ABT-199	AML	√			
	Bortezomib& Carfilzomib	MM	√	√	√	√
	Dasatinib	Ph ^+^ ALL	√		√	√

*OC* ovarian cancer, *EWS* Ewing’s sarcoma, *Ph*^*+*^*ALL* Ph^+^acute lymphoblastic leukemia, *PDAC* pancreatic ductal adenocarcinoma, *GBM* glioblastoma multiforme, *OCCC* ovarian clear cell carcinoma, *NSCLC* non-small cell lung cancer, *ER*^*+*^*BC* ER^+^breast cancer, *CRPC* castration-resistant prostate cancer, *PanNETs* pancreatic neuroendocrine tumors, *PC* pancreatic cancer, *AML* acute myeloid leukemia, *MM* multiple myeloma

**Table 3:** Combination agents of specific inhibitor of FAK

FAK Inhibitor name	Combination agents	Cancer type	In vitro experiments		In vivo experiments	
			Synergistic effects	Reverse resistance	Inhibit growth	Extended survival
Y15	Cabozantinib & sorafenib	Thyroid cancer [76]	√		√	
	4-MU	Colorectal carcinoma [77]	√		√	
	Temozolomide	Glioblastoma tumor [78]	√		√	
	PP2	Colorectal Carcinoma [91]	√		√	√
	ABT263	Lung cancer [79]	√			
	Gemcitabine	Pancreatic cancer [81]	√		√	
C4	Gemcitabine	Pancreatic ductal adenocarcinoma [83]	√		√	
	Adriamycin	Neuroblastoma [84]			√	
R2	Adriamycin & 5-FU	Colorectal carcinoma [85]	√	√		

#### Respiratory system tumors

PF573228 in combination with erlotinib reduced cell viability and tumor growth in EGFR TKI-resistant non-small cell lung cancer (NSCLC) more effective than treatment with erlotinib alone in the A549 mouse xenograft model^57^. VS-6063 in combination with Gefitinib inhibited NSCLC tumor growth both in vivo and in vitro [48]. In addition, ABT263 enhanced the efficacy of Y15, showing synergistic effects in a series of lung cancer cell lines [79].

In clinical trials, VS-6063 in combination with RO5126766 for NSCLC is in Phase I clinical recruitment, the study is designed to determine the maximum tolerated dose (MTD) and recommended Phase II dose (RP2D) of VS-6063 combined with VS-6766 in NSCLC, and commenced in December, 2017 and is expected to complete in July, 2022 [86].

#### Digestive system tumors

In pancreatic cancer, Y15 exhibited synergic anti-cancer effects with Gemcitabine [81], GSK2256098 reversed Gemcitabine-related chemoresistance [87]. C4 increased the sensitivity of tumor cells to Gemcitabine chemotherapy in vitro [83].

In pancreatic ductal adenocarcinoma (PDAC), TAE-226 in combination with Nab-paclitaxel inhibited PDAC progression and prolonged survival in hormonal mice by inhibiting cancer cell growth, invasion, and induction of apoptosis [39]. VS-6063 in combination with Nab-paclitaxel also showed synergistic effects in the treatment of PDAC [39]. Besides, PF573228 can restore cell sensitivity to lexatumumab-induced apoptosis in PDAC and showed significant inhibition of pancreatic tumor growth in xenograft mice [88]. VS-6063 synergistically performs with the mTOR inhibitor everolimus by blocking feedback AKT activation in pancreatic neuroendocrine tumors (PanNETs) [89]. Of note, VS-6063 in combination with pembrolizumab for advanced pancreatic cancer is in Phase II clinical trials (NCT03727880), the purpose of this study is to evaluate if reprograming the tumor microenvironment by targeting FAK following chemotherapy can potentiate anti-programmed death-1 (PD-1) antibody, and is expected to complete in August, 2022 [90].

In colon cancer, the combination of FAK inhibitor Y15 and the Src inhibitor PP2 reduced colon cancer cell viability more effectively than each single treatment. The combination inhibited cell growth and enhanced the efficacy of chemotherapy both in vitro and in vivo [91]. Interestingly, Y15 and the HAS inhibitor 4-methylumbelliferone (4-MU) reduced the viability of colon cancer cells in a dose-dependent manner [77]. Furthermore, R2 is able to sensitize colon cancer cells to adriamycin and 5-fluorouracil [85].

#### Bone tumors

In Ewing's sarcoma, TAE226 enhanced the efficacy of conventional chemotherapy [35]. PF-562,271 and Aurora kinase inhibitors synergistically inhibited the proliferation of Ewing’s sarcoma cells and significantly suppressed tumor progression [92].

In multiple myeloma (MM), both in vivo and in vitro, VS-4718 resensitized MM cells to the proteasome inhibitors bortezomib and carfilzomib [68].

#### Reproductive system tumors

In ovarian cancer, VS-6063 can synergistically work with paclitaxel for the treatment of advanced ovarian cancer [93]. Besides, FAK inhibition with TAE-226 re-sensitized resistant ovarian cancer cells to doxorubicin and promoted tumor regression by inhibiting angiogenesis, invasion, and inducing apoptosis levels [94]. The combination of PF562271 and ABT-737 was effective in inducing cell apoptosis in ovarian clear cell carcinoma [42]. In addition, IN10018 in combination with standard chemotherapy for high-grade serous ovarian cancer is currently in phase I clinical trial, this study was designed to evaluate the safety, tolerability and efficacy of IN10018 in combination with standard chemotherapy treatment in high-grade serous ovarian cancer, and commenced in June, 2020 [95].

On the other hand, GSK2256098 in combination with chemotherapies (paclitaxel and topotecan) showed higher sensitivity against uterine Cancer [65]. PF573228 in combination with tamoxifen was able to synergistically inhibit the proliferation of ER-positive breast cancer cells [96].

#### Other tumors

With regards to the acute leukemia, the combination of TAE226 with Nilotinib showed more significant effects than each single treatment in Ph + ALL [36]. VS-4718 exerts synergistic effects with dasatinib to Ph + B-ALL cell survival, adhesion and improved therapeutic efficacy of Ph + B-ALL in vivo [97]. In addition, VS-4718 significantly improved the efficacy of ABT-199 on inducing cell apoptosis in AML cells (including primary AML CD34 +) and AML cells overexpressing MCL-1 or BCL-XL [98].

In glioblastoma tumors, combination treatment of PF562271 and ganciclovir eliminated the implanted microglioma tumors in mice (GL261 glioma orthotopic model) [99]. Noteworthily, FAK expression and activity are elevated in brain tumor models, suggesting that FAK plays important role in brain tumor. Moreover, the combination of Y15 and temozolomide showed better outcomes than each individual treatment in vivo [78].

Furthermore, VS-6063 reversed the drug resistance of Docetaxel in castration-resistant prostate cancer [100]. Y15 was synergistic with cabozantinib, sorafenib, pazopanib, and sunitinib in treatment of thyroid cancer [76]. C4 cooperating with adriamycin shows synergistic performance in killing neuroblastoma in xenograft models [84]. These results strongly demonstrate the combination potential of FAK inhibitor in cancer therapy.

## Conclusions and future perspectives

In this review, we highlighted the impact of FAK signaling on cancer progression and elaborated the recent progress in drug development of FAK inhibitors and perspectives on FAK inhibitor therapy. As an intersection target of multiple oncogenic signaling pathways, FAK contributes to tumorigenesis and cancer progression. Accumulating research projects have demonstrated the rationality and effectiveness when employing FAK as a tumor therapeutic target. FAK inhibitors has become a hot spot in cancer drug development.

In recent years, many preclinical studies have confirmed that standard treatment supplemented with FAK-targeted drugs can significantly improve cancer prognosis and reduce chemotherapy resistance [1, 4, 101]. Thus, FAK inhibitors are going to serve as adjuvant drugs that act as chemosensitizers in cancer treatment. Although the importance of FAK inhibition is clear to the cancer therapeutic, the specific mechanism of FAK inhibitor for cancer treatment is still elusive. Further discovery is eagerly needed to elucidate how to improve the efficacy and drug resistance when apply FAK inhibitors with other anti-cancer agents. Besides, Tyr397 is the most common target site in developing FAK inhibitors, other Tyr sites are also deserved to test in the future.

On the other hand, FAK signaling pathway integrates the signal from the extracellular matrix and participates in TME remodeling in turn. Elucidation mechanism of FAK in the regulation of TME component including stroma cells (immune cell, fibroblast cells, endothelial cells) and ECM plasticity would enhance the FAK efficacy and reduce the possibility of acquired drug resistance in cancer therapy. In-depth study the function of FAK mediated signaling network between all the components will bring new ideas and chemotherapeutic strategies for the clinical treatment of tumors. Undoubtedly, highly selective FAK inhibitor combination with the standard therapies will hit cancer cells a second punch, which could benefit patients in the coming future.

Importantly, phenotypes associated with FAK inhibition show that there are multiple regulation for FAK function not only in tumor cells but also in the TME [8]. Since changes in composition and remodeling of TME are one of the most important causes in mediating tumors immune desertification, we speculate that FAK inhibitors in combination with immune checkpoint blockers such as PD-1 antibodies or CTLA-4 antibodies may exhibit impactable prospects in clinical practice. As more studies are going to be conducted in the coming years, the mechanism of FAK-related signaling pathways in the regulation of TME will also be elucidated. This will provide a more fleshed-out and rigorous scientific basis for the improvement of oncology plight.

## Data Availability

Not applicable.

## Abbreviations

AML: Acute myeloid leukemia
CAFs: Cancer associated fibroblast
CR: Complete response
CRPC: Castration-resistant prostate cancer
ECM: Extracellular matrix
ECs: Endothelial cells
ER^+^BC: ER^+^Breast cancer
EMT: Epithelial to mesenchymal transition
EOC: Epithelial ovarian cancer
EWS: Ewing’s sarcoma
FAK: Focal adhesion kinase
FERM: Four-point-one, ezrin, radixin, moesin
FAT: Focal adhesion targeting
GBM: Glioblastoma multiforme
MCL-1: Myeloid cell leukemia-1
MM: Multiple myeloma
MSC: Mesenchymal stem cell
NSCLC: Non-small cell lung cancer
OC: Ovarian cancer
OCCC: Ovarian clear cell carcinoma
OSCC: Oral squamous cell carcinoma
Ph^+^ALL: Ph^+^Acute lymphoblastic leukemia
PC: Pancreatic cancer
PDAC: Pancreatic ductal adenocarcinoma
PanNETs: Pancreatic neuroendocrine tumors
PRR: Proline-rich region
PTK2: Protein tyrosine kinase 2
RTKs: Receptor tyrosine kinases
RMS: Rhabdomyosarcoma
SCLC: Small cell lung cancer
TAM: Tumor associated macrophage
TME: Tumor microenvironment
VP: Vascular permeability
4-MU: 4-Methylumbelliferone
5-FU: 5-Fluorouracil
